# Comparing academic productivity and Instagram presence in oral and maxillofacial surgery training programs

**DOI:** 10.1007/s10006-025-01354-2

**Published:** 2025-03-04

**Authors:** Jamie Rose, Boyu Ma, Edwin M. Rojas, Jaime Castro-Núñez

**Affiliations:** 1 Department of Oral and Maxillofacial Surgery, University of Alabama at Birmingham, SDB 419, 1919 7th Ave S., Birmingham, AL 35233 USA; 2 School of Dentistry, University of Alabama at Birmingham, Birmingham, AL USA

**Keywords:** Education, Oral and maxillofacial surgery, Social media

## Abstract

**Background:**

Social media has become an increasingly important tool in how surgeons collaborate with one another, disseminate educational information, and communicate with patients.

**Purpose:**

The purpose of this study was to assess the relationship between academic productivity and social media (Instagram) presence amongst oral and maxillofacial surgery programs.

**Study design, setting, sample:**

A cross-sectional study was used to identify oral and maxillofacial surgery programs and their respective residency Instagram accounts. Information regarding number of followers, posts, and likes were recorded for each program. Academic productivity metrics for each faculty including H-index, number of publications, and number of citations were recorded.

**Predictor/exposure/independent variable:**

The independent variable was the type of residency program: certificate, dual-degree, or combined track.

**Main outcome variable(s):**

The main outcome variable was the academic influence quantified by h-index, citations, and publications of the programs and their social media influence quantified by number of followers/posts.

**Covariates:**

Instagram posts were categorized into departmental posts, educational, social, professional and miscellaneous. Engagement was further quantified by the number of likes.

**Analyses:**

Descriptive statistics, one-way ANOVA, Tukey’s Multiple Comparisons tests, ROUT’s outlier test (Q = 0.1%), and linear regression plots with a *P* value < 0.05.

**Results:**

Instagram accounts were identified for 65 (73%) of the 89 programs. There was a statistically significant moderately positive correlation between the number of followers for a program’s Instagram account compared with the number of publications (*r* = 0.5110, *P*  < 0.001). There was a statistically significant weakly positive correlation between the number of followers for a program’s Instagram account compared with average faculty h-index(*r* = 0.4982, *P*  < 0.001), and number of citations (*r* = 0.4300, *P*  < 0.001). There was a statistically significant weakly positive correlation between the number of posts for a program’s Instagram account compared with average faculty h-index (*r* = 0.3438, *P* < 0.001), number of publications (*r* = 0.3580, *P* = 0.001), and number of citations (*r* = 0.3973, *P*  < 0.001). Across all programs combined, educational posts garnered more likes compared to miscellaneous (*P* = 0.0129), social (*P* = 0.0018), departmental (*P* = 0.0005), and professional posts (*P* < 0.0001).

**Conclusion/Relevance:**

There was a moderately positive correlation between average faculty H-index and number of followers for an oral and maxillofacial surgery program’s Instagram account. There was a weak positive correlation between other measures of academic productivity and social media presence. Educational content garnered the most engagement from followers, despite surgery accounts mostly generating departmental focused posts.

## Introduction

Social media provides a platform through which surgeons can collaborate with colleagues, disseminate medical/surgical knowledge, and educate patients on a global scale [1]. In addition, many patients utilize social media as a source of information about oral and maxillofacial surgery (OMS) procedures [2]. While it has previously been established that OMS programs under-utilize web-based platforms, they are also responsible for producing high quality educational videos for patients [3, 4]. It is important as social media continues to grow in popularity that surgeons continue to stay up to date on best utilization of these platforms.

While social media use has been associated with adverse health risk behaviors, its utilization presents a unique opportunity for physicians to create a positive impact on patients’ lives through healthcare education and patient engagement [5, 6]. Others have utilized social media as a means of assessing patient satisfaction with their care [7]. Overall, previous studies have reported a positive influence of social media on patients’ health behavior [8]. Social media use by other surgical subspecialty programs, including plastic & reconstructive surgery, otolaryngology, and ophthalmology, has exploded over the past few years [9–12].

The rapid adoption of Instagram by OMS programs is clear: in June 2020 only 18.7% of residency programs had an Instagram account [13]. By January 2021, 53.3% of them had created Instagram accounts [14]. This exponential increase in social media usage was fueled by the COVID-19 pandemic and the push towards a virtual residency interview format. As a result, OMS programs are utilizing Instagram as a marketing tool towards applicants and patients alike [15]. Despite the resumption of in-person interviews, residency programs continue to utilize social media to disseminate information to their patients and communicate with prospective candidates. While others have voiced concerns over the implications of increased social media usage by surgeons, it is important to better understand how the surgeon may utilize social media to improve care and outreach [16].

Social media remains a powerful tool for career advancement [17]. Social media has allowed health professionals to advance their careers through participating in professional events and promoting expertise, products and services [18, 19]. There has been discussion about using social media influence as a factor in promotion of academic careers [20]. Other factors in the promotion of academic surgeons include academic productivity, which can be measured by the h-index [21]. The H-index, a measure of both productivity and impact, can be calculated by measuring the number of publications for which an author has been cited by other authors at least that same number of times. Academic first and senior OMS authors have an average h-index of 7.2 ± 8.4 and 13.7 ± 11.2, respectively [22].

Previous studies have shown a positive correlation between social media presence and academic productivity in other surgical specialties [23]. The purpose of this study is to assess the relationship between academic productivity and social media (Instagram) presence amongst OMS programs. Specific aims of this study include (1) to quantify the academic productivity of faculty at OMS programs; (2) to identify the most influential programs on Instagram and characterize their posts; and (3) to investigate the relationship between academic productivity and social media presence among dual degree, certificate programs, and combined track programs. The authors hypothesize this correlation holds true for OMS programs.

## Materials and methods

### Study design/sample

OMS programs on the American Association of Surgery (AAOMS) website as of 2023 were included [24]. The ten federal service programs were excluded from this study. Programs were separated into the dual-degree model, certificate model, and programs offering both tracks. The dual-degree model requires the completion of five to six years of training along with the completion of a medical degree. The certificate model requires the completion of four years of training in surgery. The name, location, type of program, and number of residency spots per year were obtained from the AAOMS website. Additional internet search was used if information on the department’s website was deemed insufficient. Faculty were identified via review of each program’s website. The following metrics of academic productivity were collected for each faculty member: h-index, number of publications, and number of citations. The bibliometric measures for the academic surgeons were computed using a bibliographic citation database (Scopus, ReedElsevier, London, UK) [25]. These indices were then averaged for the faculty at each program.

The most popular social media platforms were identified: X, Facebook, TikTok, LinkedIn and Instagram. The first 3 platforms were not searched due to minimal to no active presence of OMS programs. Only Instagram was searched due to its widespread utilization by residency programs. Utilizing the search function of the Instagram application, residency program pages were identified. Search terms such as “oms”, “oral surgery”, “surgery” and “omfs” were queried along with associated school/hospital affiliated names of each program. Residency pages were also identified via searching through followers of society accounts and other residency program accounts. Residency program accounts were verified to filter out accounts operated by individuals. The handle, number of followers, following, and posts associated with each account were recorded.

The five most recent posts if applicable were categorized into the following: educational, departmental, academic/professional, social, and miscellaneous. Educational posts included any posts with content related to surgical techniques, pathology, operative imaging, surgical planning, and hardware. Departmental posts included department-related information, promotions, department-sponsored community outreach/international trips, and resident/faculty/staff highlights. Academic/professional posts included content regarding resident/faculty research, academic meetings and conferences, lectures, and professional events hosted by third parties. Social posts included any posts related to resident life or events occurring outside of the workplace. All other posts were categorized as miscellaneous. Posts were further subcategorized into pictures and videos (“reels”). The number of likes for each post were recorded. Accounts with fewer than five posts and private access to the number of likes per post were excluded from the data set. For posts containing multiple pictures/reels, only the first picture/reel and the caption were utilized to categorize the post.

### Statistical analysis

**Fig. 1:**
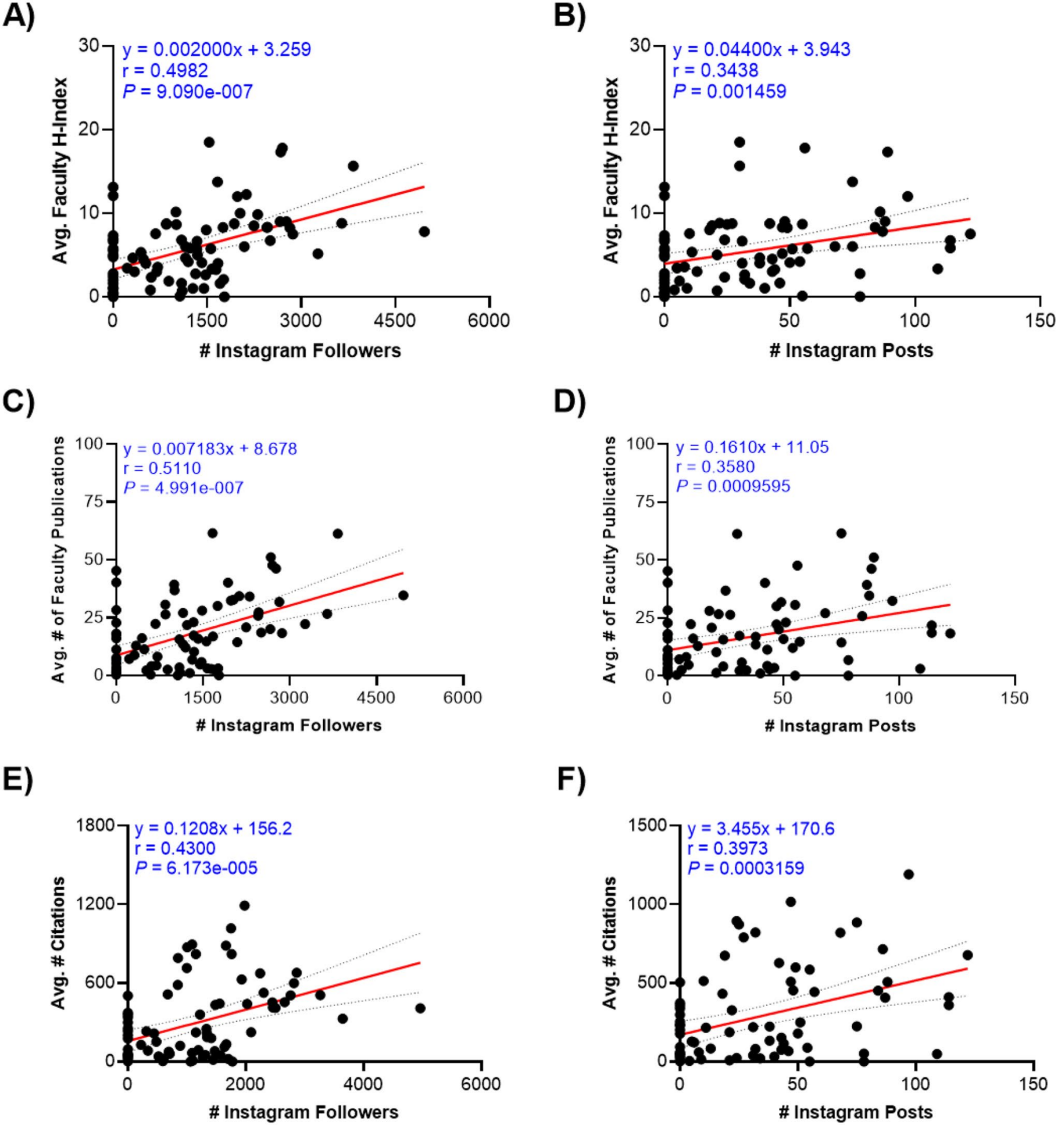
**A-F** Scatterplots providing a visual representation of logistic regression analysis of the Instagram followers and posts compared to faculty H-index, publications, and citations. Black dots represent individual programs, areas encompassed by gray represent the 95% confidence interval, and lines of best fit are given in red

The primary outcome of interest, academic productivity, was measured by H-index, number of publications, and number of citations for each faculty. Descriptive statistics were utilized to characterize the social media presence of OMS programs of dual-degree, certificate, and combined track programs. Univariate ANOVA analyses were used to determine significant differences with respect to academic productivity measurements amongst the faculty of these programs (Table 1). Next, the average h-index, number of publications, and number of citations among faculty members for each program were calculated. Univariate (ANOVA) analyses were used as appropriate to assess social media presence and academic productivity of dual-degree versus certificate versus combined track programs (Table 1). A series of linear regressions and scatter plots were generated to evaluate the relationship between social media presence and academic productivity (Fig. 1). Significant findings from these linear regression plots are summarized in Supplemental Table 1.

**Table 1:** One-way ANOVA analysis of Instagram and academic characteristics among faculty affiliated with certificate, dual-degree, and combined track OMS residency programss

	Certificate *N* = 279 Mean (± SD)	Dual-degree *N* = 175 Mean (± SD)	Combined *N* = 155 Mean (± SD)	*P* value
Residency Program Instagram accounts (n, %)	30, 63.8%	18, 78.2%	17, 89.5%	—
Total publications	9.860 (22.63)	29.19 (46.34)	23.02 (37.54)	< 0.0001
H-Index	3.437 (5.201)	8.920 (11.88)	7.103 (9.521)	< 0.0001
Total citations	156.1 (401.6)	816.1 (1933)	523.1 (1184)	< 0.0001

Microsoft Excel (Redmond, WA) was used to quantify, organize, and secure the data between the researchers. Data from Microsoft Excel was transferred to GraphPad Prism 10.2.0. (La Jolla, CA). Statistical analyses, including descriptive statistics, one-way ANOVA, Tukey’s Multiple Comparisons tests, ROUT’s outlier test (Q = 0.1%), and plots were generated and analyzed with GraphPad Prism. *P* values < 0.05 were considered statistically significant. Error bars denote standard deviation (SD). All of the Instagram data was collected by one of the author’s new Instagram accounts from January 8-15th, 2024. An IRB was not required as the data we collected on Instagram was freely accessible to the public.

## Results

### Data collection

Review of the AAOMS and program websites identified 606 faculty representing 89 OMS programs. Of these, 279 were associated with certificate programs, 175 were associated with dual-degree programs, and 155 were associated with combined programs. Instagram accounts were identified for 65 (73%) programs.

### Association between faculty academic metrics and social media presence

There was a statistically significant moderately positive correlation between the number of followers for a program’s Instagram account compared with the number of publications (*r* = 0.5110, *P*  < 0.001). There was a statistically significant weakly positive correlation between the number of followers for a program’s Instagram account compared with average faculty h-index (*r* = 0.4982, *P* < 0.001), and number of citations (*r* = 0.4300, *P* < 0.001) (Fig. 1a,c,e). There was a statistically significant weakly positive correlation between the number of posts for a program’s Instagram account compared with average faculty H-index (*r* = 0.3438, *P*< 0.001), number of publications (*r* = 0.3580, *P* = 0.001), and number of citations (*r* = 0.3973, *P* < 0.001) (Fig. 1b,d,f). A summary of these statistically significant findings may be found in Supplemental Table 1.

**Fig. 2:**
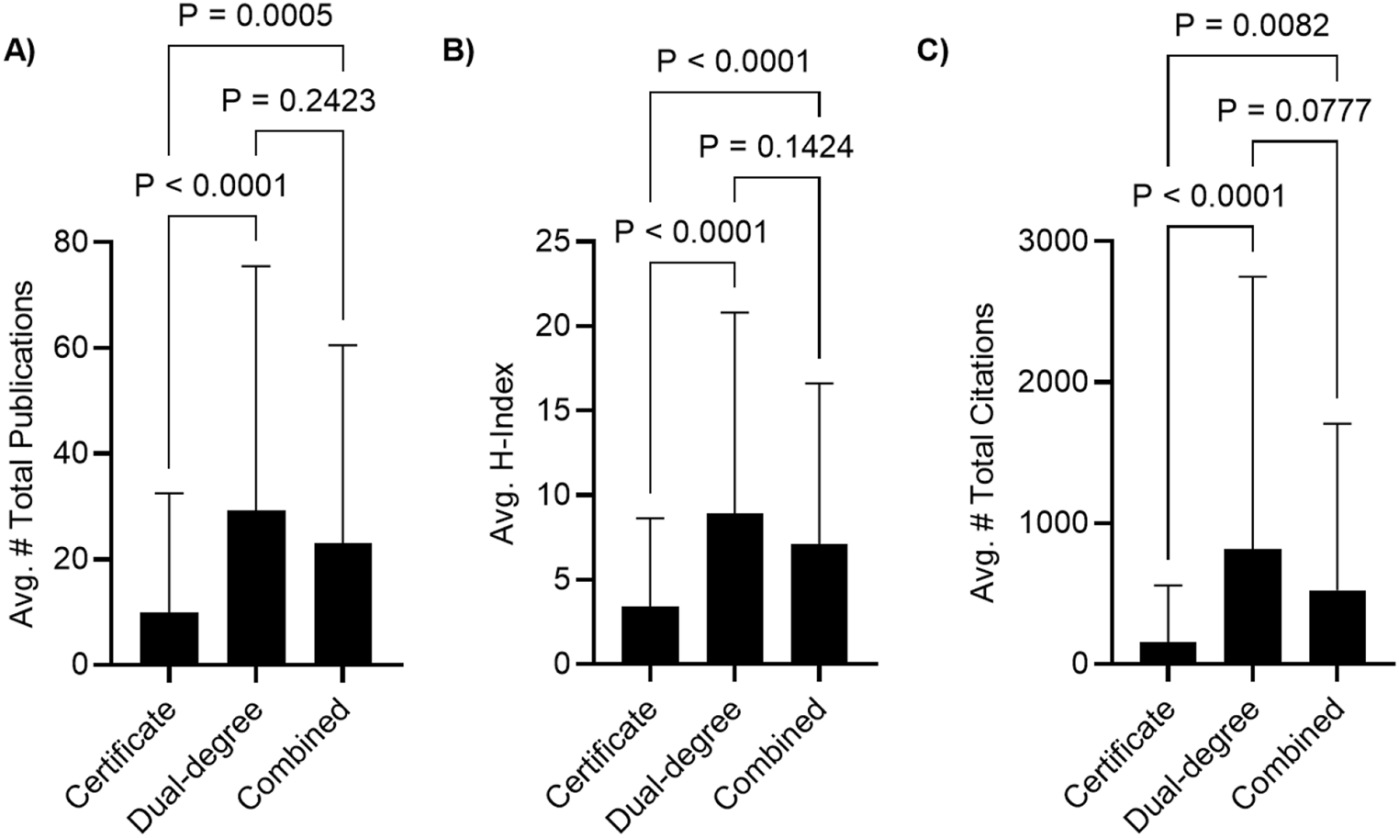
**A-C** Bar graphs comparing academic productivity metrics amongst OMS residency programs: certificate, dual-degree, and combined track. (**A**) The average number of total publications was compared between programs. (**B**) The average H-index was compared between programs. (**C**) The average number of total citations was compared between programs. Statistical analysis was performed by one-way analysis of variance (ANOVA) and Tukey’s Multiple Comparisons tests. *P* values are listed above the brackets. Error bars denote standard deviation (SD)

### Faculty and program characteristics

There was no statistically significant difference between presence of a residency Instagram account between certificate programs (30, 63.8%), combined track programs (18, 78.2%), and dual-degree programs (18, 78.2%). (See Table 1). Faculty of dual-degree or combined track program was associated with higher H-index (8.920 ± 11.88; 7.103 ± 9.521 vs. 3.437 ± 5.201, *P* < 0.0001), number of publications (29.19 ± 46.34; 23.02 ± 37.54 vs. 9.860 ±22.63, *P* < 0.0001), and number of citations (816.1 ± 1933; 523.1 ± 1184 vs. 156.1 ± 401.6, *P* < 0.0001) (See Table 1; Fig. 2a-c). There was no statistically significant difference between faculty H-index, number of publications, or number of citations between combined track programs and dual degree programs.

### Characterization of social media content

**Fig. 3:**
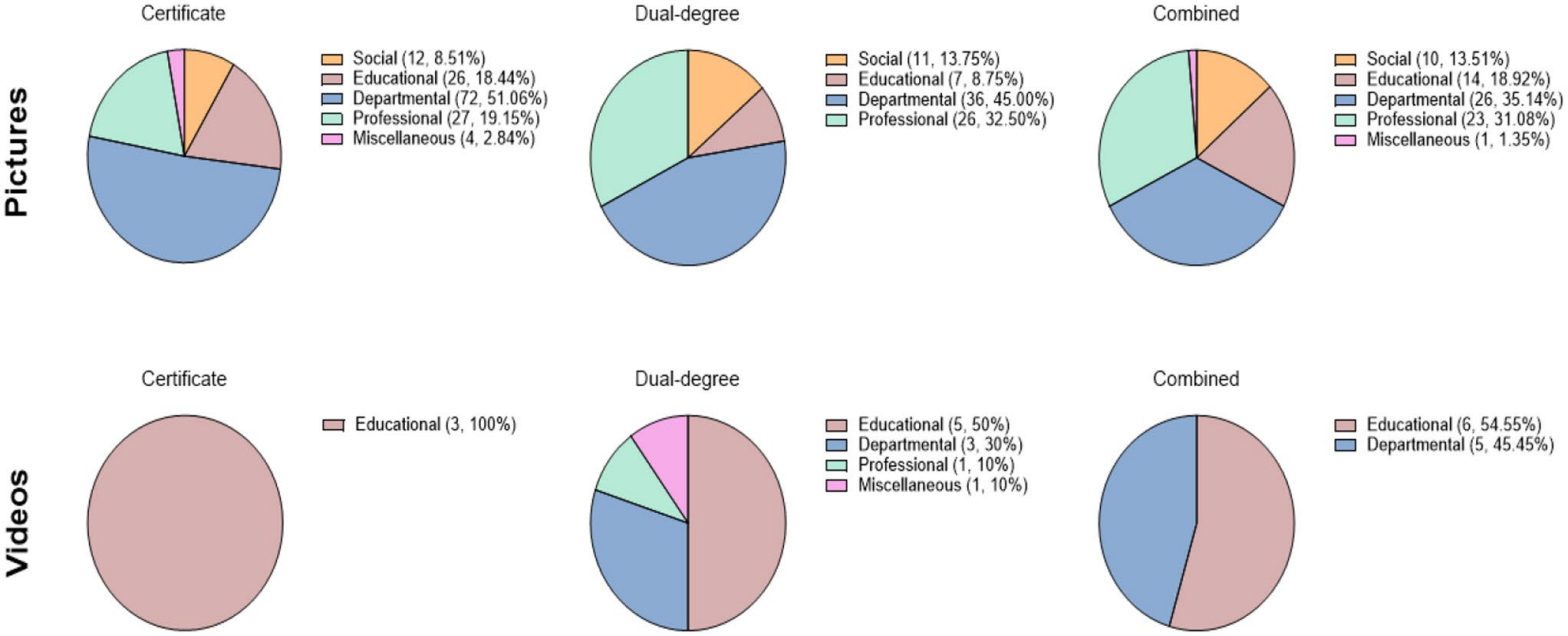
Pie charts representing the Instagram content (pictures and videos) posted by certificate, dual-degree, and combined track OMS programs. Categories of pictures and videos include social, educational, departmental, professional, and miscellaneous.

Departmental posts accounted for 45% of dual-degree program posts, 35% of combined track program posts, and 51% of certificate programs. The distribution of social media content by program type is summarized in Fig. 3a-f.

**Fig. 4:**
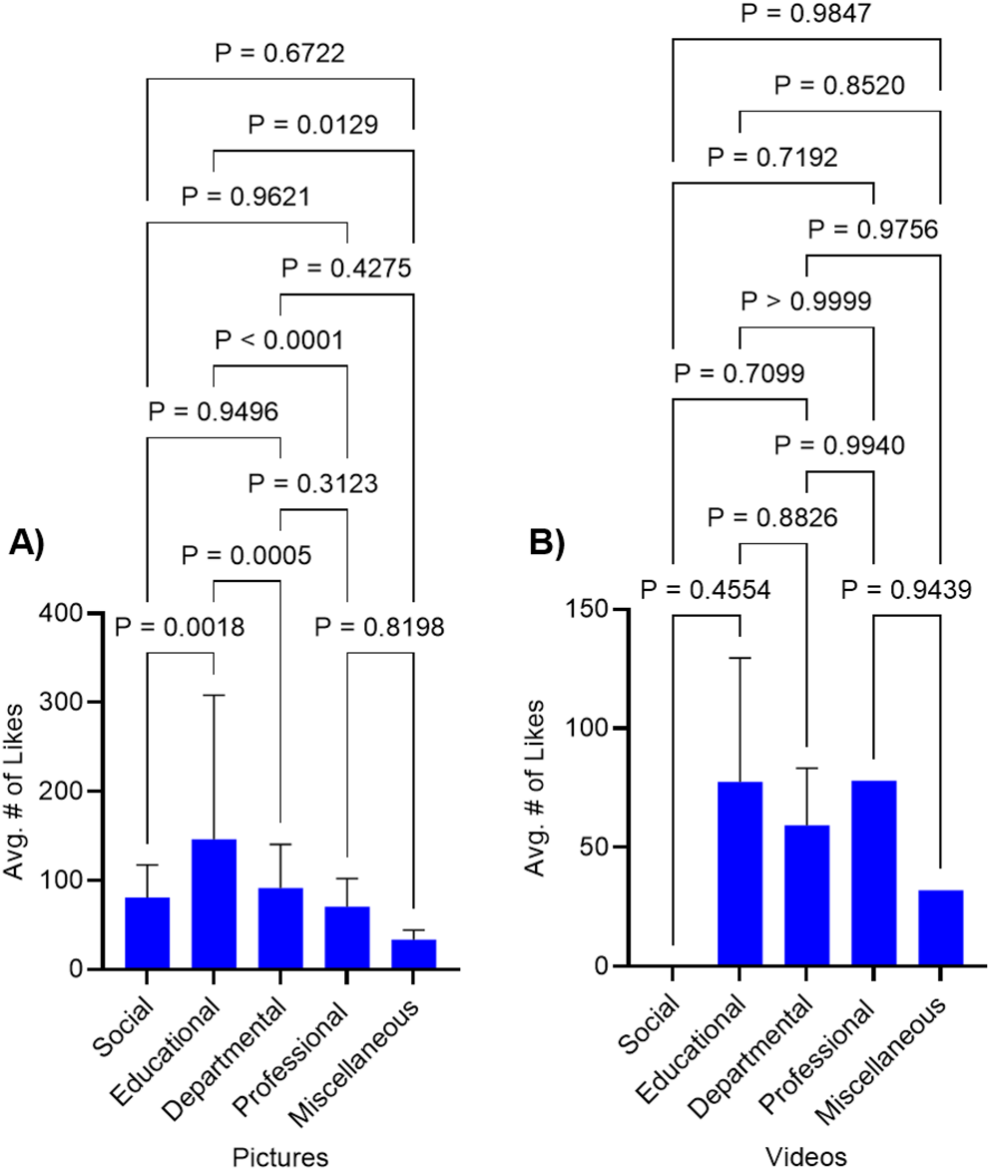
**A-B** Bar graphs comparing the average number of likes between types of content (**A**: pictures; **B**: videos) posted by residency programs on Instagram. Categories include social, educational, departmental, professional, and miscellaneous. Statistical analysis was performed by one-way analysis of variance (ANOVA) and Tukey’s Multiple Comparisons tests. *P* values are listed above the brackets. Error bars denote standard deviation (SD).

Across all programs combined, educational posts garnered more likes (146.02 ± 85.85) compared to miscellaneous (33.60 ± 10.4, *P* = 0.0129), social (80.90 ± 36.63, *P* = 0.0018), departmental (91.48 ± 48.94, *P* = 0.0005), and professional posts (70.41 ± 31.64, *P* < 0.0001) (See Fig. 4). There was no statistically significant difference between the number of likes received by miscellaneous, social, departmental, or professional posts. The sample size of video posts was too small to make any statistically significant conclusions.

## Discussion

Social media usage amongst OMS programs has expanded over the last several years to become an effective communication tool for the dissemination of medical knowledge, patient outreach, and education. The purpose of this study was to analyze the academic productivity of OMS faculty, evaluate the most influential programs on Instagram and characterize their posts, and assess the relationship between academic productivity and social media presence amongst OMS programs. To the authors’ knowledge, this is the first study assessing the relationship between academic productivity and social media presence of OMS programs. More residency programs than ever before are engaging patients, colleagues, and potential applicants through Instagram with 73% of programs boasting a residency Instagram account compared to 48% in 2021 [13, 14]. Departmental focused posts accounted for the largest category of posts across all program types. In contrast, educational content proved to garner far more engagement from followers than any other type of post. Previous studies looking at the utilization of TikTok as an educational tool demonstrated a need for oral health professionals to create accurate educational content [3]. Overall, this highlights an opportunity for productive academicians to share medical knowledge/educating patients and colleagues.

As OMS residency programs, residents, and attendings continue to utilize this tool to engage with patients and colleagues, it is important for surgeons to keep in mind the medicolegal implications of their posts. Laws governing the use of patient information in social media vary from country to country. OMS surgeons must go to great lengths to protect patient health information whenever possible. This proves exceedingly difficult in the case of the OMS surgeon as much of our work revolves around the face. It is advisable to post content through dedicated professional accounts rather than personal accounts if possible.

Faculty associated with dual-degree and combined track programs were more likely to have a higher H-index, more publications, and more citations compared to faculty from certificate programs. The aforementioned results beg the question: why do the faculty associated with dual-degree and combined track programs have higher academic indices compared to their certificate program counterparts? Part of the answer to this question may be that dual-degree and combined track programs benefit from the backing of academic institutions and medical schools with large NIH funding that may provide more resources for researchers [26]. Academic institutions may have greater criteria for promotion and tenure which include publications to reach the next level of professorship [27]. This would generate more incentive for publishing academic content. In addition, the additional two years of training in most dual-degree programs provides residents with more opportunity and time to collaborate with faculty and contribute to the academic literature [28]. While we acknowledge that we cannot formulate conclusions about why exactly there is a difference in academic productivity, future projects should be developed to investigate the disparity in research between certificate and dual-degree OMS programs.

This study found a moderately positive correlation between average faculty H-index and number of followers for an oral and maxillofacial surgery program’s Instagram account. There was statistically significant weakly positive correlation between other measures of academic productivity and social media presence. Although there is a correlation, we are unable to determine whether academic productivity improves social media presence or visa versa. Large academic institutions with high research output benefit from increased personnel and financial support for social media outreach compared to smaller hospital-based programs [28]. Currently only 8.1% of allopathic institutions stated in a study in 2021 that they would use digital and social media products for scholarship and promotion [29]. Social media influence could potentially be a new avenue of digital scholarship.

There are several limitations in this study. Firstly, social media analysis was limited to Instagram. Although other social media platforms such as X, Facebook, LinkedIn and TikTok exist, the authors chose to focus on Instagram presence due to its rapid adoption by OMS residency programs [13]. These social media sites are increasingly attracting the attention of physicians intrigued by their potential and reach. There are over 3 billion Facebook users, 600 million X users, 2 billion TikTok users, and 1 billion LinkedIn users [30–33]. LinkedIn in particular serves an important role as an important means for professional networking. The growing popularity of TikTok in recent years represents the ever evolving landscape of social media. Excluding data from these other social media sites represents a potential gap in possible avenues of social media engagement.

In addition, this study was unable to assess engagement via the Instagram “story” function. The story function allows Instagram users to create temporary posts, typically viewable for less than 24 hours by followers. As this study was conducted primarily on United States OMS residency programs, the results may not be generalizable to programs in other countries. The academic metrics and social media data represented a specific snapshot in time and did not account for ongoing rapid changes or these temporary posts. Additionally, program websites were not uniform in their listing of faculty. Some programs did not include or delineate part time clinical faculty from full-time faculty on their websites.Retirement, change of institution or department, or the manner a name is spelled out or abbreviated may affect the h-index.

In conclusion, this study investigated the relationship between academic productivity and social media presence amongst OMS programs and their faculty. The authors found a moderately positive correlation between average faculty H-index and number of followers for an oral and maxillofacial surgery program’s Instagram account. There was a statistically significant weakly positive correlation between other measures of academic productivity and social media presence. Future studies may be warranted to explore factors that may contribute to this relationship. The increasing popularity of social media provides an opportunity for academic oral and maxillofacial surgeons to expand their academic influence, collaborate with other scholars, educate the public, and disseminate their ideas.

## Data Availability

No datasets were generated or analysed during the current study.
